# Advancing the Understanding of the Factor Structure of Executive Functioning

**DOI:** 10.3390/jintelligence9010016

**Published:** 2021-03-16

**Authors:** Samsad Afrin Himi, Markus Bühner, Sven Hilbert

**Affiliations:** 1 Department of Psychology, Jagannath University, Dhaka 1100, Bangladesh; 2 Psychological Methods and Assessment, Department of Psychology, Ludwig-Maximilians-Universität München, Leopoldstraße 13, 80802 Munich, Germany; buehner@lmu.de; 3 Educational Science and Sport Science, Faculty of Psychology, University of Regensburg, Universitätsstraße 31, 93053 Regensburg, Germany

**Keywords:** executive functioning, factor structure, task impurity, working memory capacity, relational integration, divided attention

## Abstract

There has been considerable debate and interest regarding the factor structure of executive functioning (EF). Therefore, the aim of the current study was to delve into this issue differently, by investigating EF and other cognitive constructs, such as working memory capacity (WMC), relational integration, and divided attention, which may contribute to EF. Here, we examined whether it is possible to provide evidence for a definite model of EF containing the components of updating, shifting, and inhibition. For this purpose, 202 young adults completed a battery of EF, three WMC tests, three relational integration tests, and two divided attention tests. A confirmatory factor analysis on all the cognitive abilities produced a five-factor structure, which included one factor predominately containing shifting tasks, the next factor containing two updating tasks, the third one predominately representing WMC, the fourth factor consisting of relational integration and antisaccade tasks, and finally, the last factor consisting of the divided attention and stop signal tasks. Lastly, a subsequent hierarchical model supported a higher-order factor, thereby representing general cognitive ability.

## 1. Introduction

Executive functioning (EF) continues to be an interesting topic of investigation regarding its cognitive underpinning and debate of relevant assessment procedures. EF can be referred to as an “umbrella term”, and is synonymous with the terms “cognitive control” or “attentional control”, as it is concerned with the control of goal-directed behavior (von Bastian et al. 2020). EF can be either characterized as a unitary cognitive construct or as representing a diverse set of functions. Therefore, some researchers have taken the approach of using several measures for distinct components of EF (Fleming et al. 2016; Friedman et al. 2016; Ito et al. 2015), whereas others use several measures to assess a single EF component (Ettenhofer et al. 2006). As such, there has been a longstanding debate about the “elusive nature” or “task-impurity problem” of EF for decades, as well as discussion about the relationship between the tasks. As described by Snyder et al. (2015), this problem makes interpreting the results difficult because the amount of variance attributed to unique, as well as common, EF variance can be relatively small compared to non-EF variance.

To address this task impurity problem, the most-cited, as well as widely lauded, seminal work by Miyake et al. (2000) proposed an interrelated three-factor model, consisting of “shifting between the task sets”, “updating the content in working memory”, and “inhibiting the pre-potent response”. Later, Friedman et al. (2016) replicated this EF model on young adults. Although the confirmatory approach of this model has burgeoned, the solution has varied significantly across studies. For instance, the studies conducted with young adults: (1) identified either two (Klauer et al. 2010), four (Chuderski et al. 2012), or five-factor solutions (Fournier-Vicente et al. 2008); (2) used different task sets for measuring the same EF component (Fournier-Vicente et al. 2008); and (3) showed insufficient indicators per latent constructs (Klauer et al. 2010). Moreover, besides Friedman et al. (2016), Ito et al. (2015), Fleming et al. (2016), and Himi et al. (2019), no other researchers have tried to replicate the EF models using similar task sets in samples of young adults.

Interestingly, the ongoing controversy on the structure of EF also exists in the early childhood studies (Morra et al. 2018). For example, Im-Bolter et al. (2006) proposed a two-layer, four-factor model, in which two attentional components, specifically mental attentional and interruption capacity, include two EF—updating and shifting. Agostino et al. (2010) provided further support for this model. Together, these have added further complexity to the debate on the EF structure. Therefore, we designed the current study to expand the model beyond the evaluation of three core EF factors. We included other posited constructs—working memory capacity (WMC; representing the storage and processing/complex span task), relational integration, and divided attention.

### 1.1. Relationship between EF and Relevant Cognitive Constructs

Our interest in EF factor structure stemmed from a desire to understand if EF is influenced by other cognitive constructs. Evidence that WMC, relational integration, and divided attention are substantially related to EF was demonstrated by Himi et al. (2019), thus reflecting an overlapping domain-general executive process. The conceptualization of WMC and EF might have a considerable impact on our theoretical understanding of these two constructs. On a neuronal level, the prefrontal cortex reflects similar activity between these constructs (Miyake et al. 2000; Osaka et al. 2003), despite the fact that distinct tasks are used for each construct. Therefore, it can be assumed that the task that has been used to assess WMC and EF taps a common underlying inhibitory control ability that is related to higher-level cognition (e.g., McCabe et al. 2010).

To assess relational integration (a paradigm-specific working memory factor; Oberauer et al. 2007), we used the measures of “monitoring” or “integrating” of related actions. Monitoring is considered as an EF ability (Gathmann et al. 2017), as it describes the capacity to update and keep track of information while processing several tasks. Patients with Alzheimer’s disease perform poorly on relational integration measures, as well as display evident deficits in EF tasks (Waltz et al. 2004).

The other component that we investigated was divided attention, which is inhibitory in nature (Kane and Engle 2000). The tasks usually used to measure interference control abilities typically assess divided attention by focusing on relevant information and ignoring irrelevant information. Consequently, there is a need to understand the way that WMC, relational integration, and divided attention relate to the more usual assessment of EF. Factor analysis provides us an insight into this regard. In accordance with the previous studies, confirmatory factor analysis was performed, as the applied tasks are more or less related to each other.

### 1.2. The Current Study

Our investigation into the factor structure of EF in adults was carried out on data collected from one of our previous studies (Himi et al. 2019). The present research was designed to address the following research question: Do factor analyses using EF abilities and the inclusion of other basic cognitive skills (a combination of WMC, relational integration, and divided attention) modify the EF factor structure as proposed by Friedman et al. (2016)? To test this, we used identical EF test battery and scoring systems as followed by Friedman et al. (2016).

## 2. Methods

### 2.1. Participants

A total of 202 younger adults between the ages of 17 to 35 years (73.3% women, mean age = 23.09 years, *SD* = 3.86 years) participated in this study. All participants had normal or corrected-to-normal vision and hearing. All of them were rewarded with either a certificate of participation in an empirical study or a payment of €50 after completion.

### 2.2. Measures

We used three tasks each (reflecting verbal, numerical, and figural) to measure all cognitive constructs (except inhibition and divided attention). All EF tasks (shifting, updating, and inhibition) were replicated in the same way as by Friedman et al. (2016), except for the stopsignal and the nonverbal *n*-back tasks because of the unavailability of the original tasks—(we also collected the Stroop task data but did not consider this in the study). A short description of each task is provided in Table 1. A more detailed description of the tasks can be found in Himi et al. (2019).

### 2.3. Procedures

Written informed consent was obtained from all participants prior to data collection. The study was conducted in two sessions on separate days within a period of one to two weeks, lasting about three hours each, including a ten-minute break. The participants were tested either individually or in a group setting in a university laboratory. Detailed procedures were given in Himi et al. (2019).

## 3. Statistical Analyses

### 3.1. Data Trimming and Transformation

Observations falling above ±3*SD*s from the mean of each group were replaced by the values equal to ±3*SD*s from the mean. Raw scores of the variables (except for the nonverbal *n*-back task) were used for all analyses. Regarding nonverbal *n*-back, Friedman and her collogues used the arcsine transformed score instead of raw score. We used a similar scoring system for this task to parallel the analysis of Friedman et al. (2016). Additionally, the scores of all RT measures were recorded inversely, so that higher scores represented higher performance.

### 3.2. Data Analyses

Parallel analysis and exploratory factor analysis were conducted using the open-source statistical software R (R Development Core Team 2020). The “psych” package (Revelle 2020) was used for the parallel and the exploratory factor analysis. The estimated models were tested by confirmatory factor analysis. The assessment of the global goodness-of-fit was based on a Chi-square test (χ^2^), the standardized root mean square residual (SRMR), the root mean square error of approximation (RMSEA), and the comparative fit index (CFI). Values of SRMR < .08, RMSEA < .06, and CFI > .95 were taken as indication of adequate model fit (Hu and Bentler 1999). We also used Akaike information criterion (AIC) and Bayesian information criterion (BIC) while comparing between the models. A smaller AIC or BIC score favors a better model. We reported standardized loadings of each indicator on its corresponding latent factor. All models were estimated using Amos 24.

## 4. Results

### 4.1. Preliminary Data Analysis

Means, standard deviations, and reliability estimates for all measures are presented in Table 2. The reliability estimates showed mostly high and consistent results with the literature, with a few exceptions (i.e., the letter memory, stop signal, and symmetry span tasks). In the beginning of the analyses, we reviewed the model displayed in Himi et al. (2019, Figure 2) to understand how all basic cognitive abilities overlapped with one another. This model showed that all factor loadings differed significantly from zero, except for the Stroop task. A likely reason for this is that the Stroop task was based on manual responses, rather than verbal responses. As shown in Hilbert et al. (2014), this may lead to a disappearance of the desired effect. Therefore, we did not include the Stroop task in the present study. Furthermore, Himi et al. also demonstrated that the latent factors were correlated with each other.

Critically, foremost, before going to our final factor analysis, we performed a preliminary analysis to understand the factor structure within the EF variables. Therefore, a measure of sample adequacy (MSA) for the exploratory factor analysis was examined with the Kaiser–Meyer–Olkin (KMO) index, which showed an overall MSA value of .71. The results yielded a two- or four-factor structure with respect to the scree plot and parallel analysis, respectively. Although the scree plot is very subjective, we decided to retain the two-factor solution, as in the four-factor solution, the two inhibition tasks loaded alone onto different latent factors. However, in a two-factor structure (see Appendix A) updating and inhibition tasks loaded onto a single variable, as well as the shifting tasks predominately loaded on an individual factor.

### 4.2. Five-Factor Model

**Figure 1:**
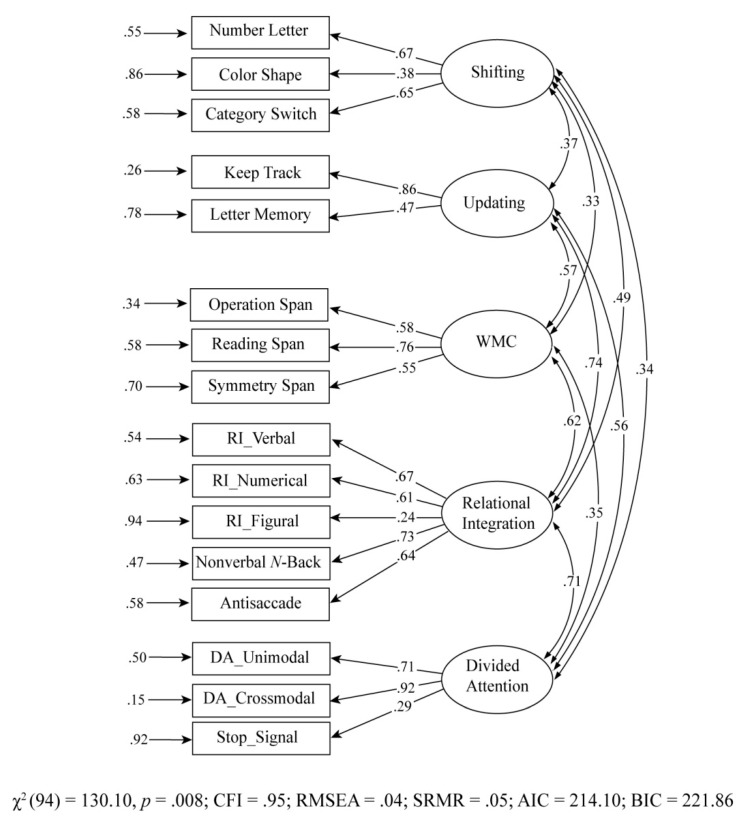
Correlated cognitive latent variables models. The proportion of residual variance of each indicator was calculated by subtracting the variance of the indicator from 1. All parameters were statistically significant (*p* < .05). WMC = working memory capacity; RI = relational integration; DA = divided attention.

From a theoretical as well as an empirical point of view, using exploratory factor analysis, Himi et al. indicated a five-factor structure of EF (see supplementary analysis in Himi et al. 2019): updating, shifting, inhibition, WMC, relational integration, and divided attention. Unlike Friedman et al. (2016), their EF model did not show a three-factor structure. Rather, the loadings were distributed amongst the five extracted factors. The shifting tasks alone loaded on a separate factor, whereas inhibition and updating tasks were distributed onto different latent factors. Based on these extracted factors, we tested a five-factor EF model using a confirmatory factor analysis with correlated latent variables. Figure 1 represents the applied measurement model. The fit of the model was adequate, χ^2^(94) = 130.10, *p =* .008; CFI = .95; RMSEA = .04; SRMR = .05, AIC = 214.10; BIC = 221.86. All path coefficients from the indicators to the corresponding latent variables in this model were moderate to high (shifting: λ = .38 to λ = .67; updating: λ = .47 to λ = .86; WMC: λ = .55 to λ = .76; relational integration: λ = .24 to λ = .73; divided attention: λ = .29 to λ = .92), and were all significant (all *p* < .01). Correlations between the latent variables were also moderate to high (ranging from *r* = .37 to *r* = .74). The shifting factor shared the lowest variance with the other constructs, whereas relational integration and updating shared 55% of the variance.

### 4.3. Hierarchical Model

**Figure 2:**
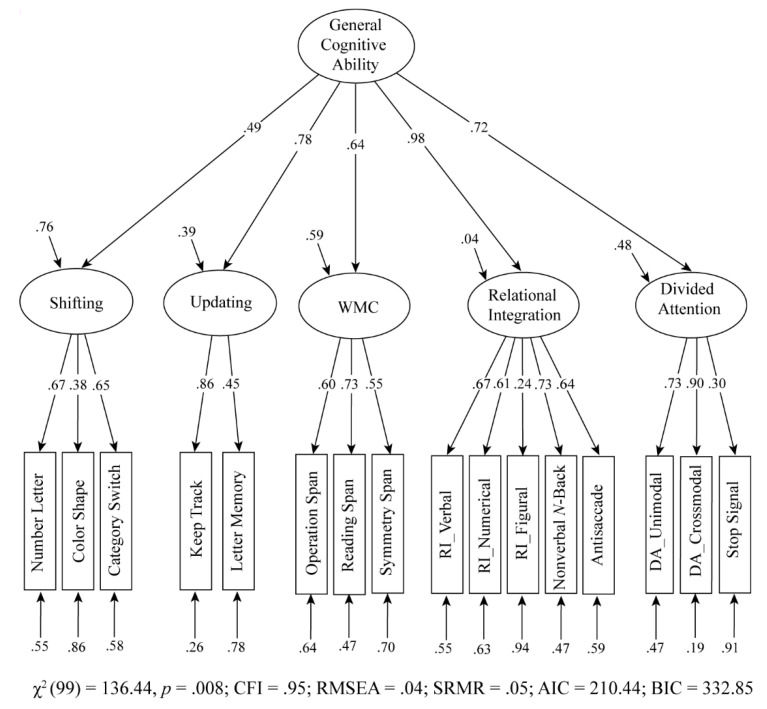
Hierarchical model of cognitive latent variables. The proportion of residual variance of each indicator was calculated by subtracting the variance of the indicator from 1. All parameters were statistically significant (*p* < .05). WMC = working memory capacity; RI = relational integration; DA = divided attention.

The correlational findings between the latent variables raise the question as to whether these factors reflect more general cognitive ability. Using a hierarchical model (Figure 2), the variance in the five cognitive latent variables was split between two components, what was shared between the latent variables and what was unique. All latent variables loaded significantly on the higher-order factor. This hierarchical model fitted the data well, χ^2^(99) = 136.44, *p =* .008; CFI = .95; RMSEA = .04; SRMR = .05; AIC = 210.44; BIC = 332.85. General cognitive ability accounted for 24% of the shifting variance, 61% of the updating variance, 41% of the WMC, 96% of the relational integration, and 52% of the divided attention variance. Thus, there was both significant shared variance for the five cognitive variables, as well as significant unique variances on each of the cognitive abilities.

### 4.4. Additional Model: Two-Layer Six-Factor Model

**Figure 3:**
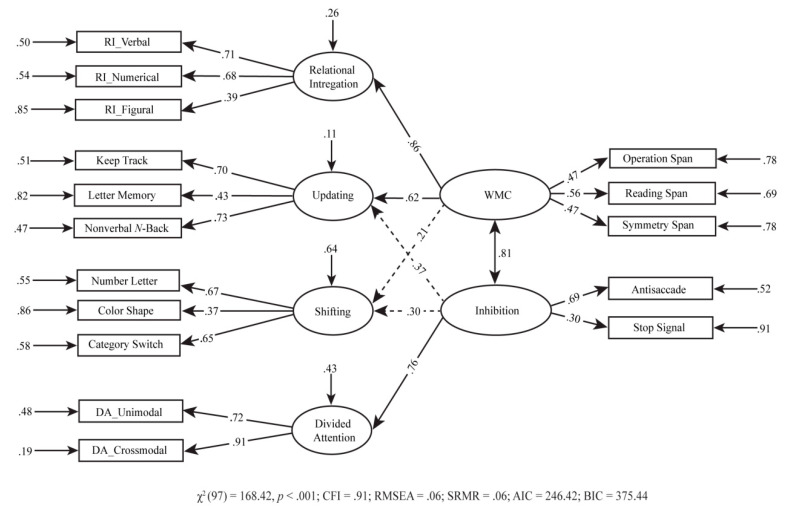
Two-layer six-factor model. The proportion of residual variance of each indicator was calculated by subtracting the variance of the indicator from 1. Not significant (*p* < .05) paths are indicated as the dotted line. WMC = working memory capacity; RI = relational integration; DA = divided attention.

Finally, motivated by Im-Bolter et al. (2006), we intended to test their four-factor model on the adult sample. We extended Im-Bolter et al.’s model without modifying its core structure, by including relational integration and divided attention. The fit of the resulting model (Figure 3) was adequate, χ^2^(97) = 168.42, *p* < .001; CFI= .91; RMSEA= .06; SRMR= .06; AIC = 246.42; BIC = 375.44. All path coefficients in this model were significant, except for the paths from inhibition and WMC to shifting and from inhibition to updating. The correlation between WMC and inhibition was high (*r* = .81). Notably, the relational integration factor seemed to be almost isomorphic with WMC (λ = .86).

### 4.5. Model Comparison

Considering the five-factor model as a baseline model, we compared the relative fits of the hierarchical model and the two-layer six-factor model. As presented in Table 3, the five-factor model showed a significantly better fit than two-layer six-factor model (∆χ^2^(3) = 38.32, *p = .*0001) and an equally good one compared to the hierarchical model (∆χ^2^(5) = 6.34, *p =* .275). However, the hierarchical model is more parsimonious, as underlined by the information criteria (AIC and BIC).

## 5. Discussion

A closer inspection into the factor structure of EF casts doubt onto the original model proposed by Friedman et al. (2016), as our findings did not replicate that of the original model. In our model, we further investigated the EF structure by expanding the model beyond the evaluation of three EF factors that is including other posited constructs (WMC, relational integration, and divided attention). Although we used identical EF test battery and scoring systems as Friedman et al. (2016), the results did not identify a definitive measurement model of EF in aggregate. These findings were thus inconsistent with the EF structure of the prior studies (Friedman et al. 2016; Miyake et al. 2000). It was only found that indicators of shifting loaded significantly on the latent shifting factor, but the *n*-back task and the indicators of inhibition either loaded on relational integration or divided attention. However, it needs to be kept in mind that the data of the Stroop task were not considered in this study.

The analysis on the eight EF variables using exploratory factor analysis primarily extracted the two-factor solution, in which inhibition and updating factors loaded onto a single component and shifting tasks loaded on the shifting-specific component (see Appendix A), making shifting the only clearly isolatable component of the proposed EF structure. Moreover, the reason why inhibition and updating were merged into one factor (similar to Adrover-Roig et al. 2012; Klauer et al. 2010) was that the two constructs depend on each other. Updating requires inhibition to successfully disengage irrelevant information and to reduce interference in and around the focus of attention (Cowan 2001; Oberauer et al. 2007). Thus, the overlapping variance of updating and inhibition appears to support the organization of memory and attention around encoding limited relevant information strongly, rather than a lot of information weakly. Notably, Panesi and Morra (2020) recently found a similar factor structure in children. On the other hand, the shifting tasks purely reflect the EF ability, meaning it reflects the ability to flexibly switch between task sets.

Further analysis was conducted with the inclusion of nonexecutive cognitive tasks—WMC, relational integration, and divided attention. This inclusion resulted in a different factor structure, which was tested by confirmatory factor analysis (Figure 1). The updating and inhibition tasks loaded differently onto the different components, although the tasks appeared to measure shifting, WMC, relational integration, and divided attention, which loaded onto their skill-specific corresponding latent variables. The updating task—nonverbal *n*-back task—loaded on relational integration. The nonverbal *n*-back task requires one to identify the stimulus that matches the stimulus *n-*times before. The *n*-back task calls for a high level of monitoring demand, as it requires one to integrate stimuli through established relationships of the stimuli, rather than simply updating the information in memory. Additionally, the inhibition tasks—stop signal and antisaccade—loaded on divided attention and relational integration, respectively. Together, these results support the view of domain-general central processing resources (Im-Bolter et al. 2006).

Moreover, the results of the hierarchical cognitive model (Figure 2) showed a large amount of overlapping variance across all five cognitive abilities, which reflects the general cognitive ability, as described by the common cognitive latent variable. The idea that cognitive measures share common variance, referred to as “g” has a long history in psychometrics (Spearman 1904), although the inclusion of a g factor is controversial because of its lack of metric invariance (Horn and McArdle 2007). In this regard, it may be argued that the hierarchical g accounts for the correlations among the first-order factor model, but not the correlations among the manifest indicators (Kovacs and Conway 2016), unlike Spearman’s original view. Therefore, the first-order factors showed a positive correlation and a good model fit in the correlated five-factor model of the current study (Figure 1). Moreover, each first-order latent factor was derived from the sample of verbal, numerical, and figural content tests. However, the common cognitive latent variable (the sum of all cognitive abilities) predicted the latent shifting, updating, WMC, relational integration, and divided attention, thus contrasting the assumption of the process overlap theory (Kovacs and Conway 2016). In other words, general ability seemsto be a source of individual differences in all cognitive abilities. However, relational integration showed the strongest connection to general ability, compared to other constructs. This suggests that relational integration shared the greatest variance with all other cognitive abilities. This was modeled by the higher-order factor, which loaded almost perfectly on relational integration. According to Halford et al.’s (1998) theory, the ability to process complex relations contributes largely to cognitive development and one may hypothesize that the same holds true for individual differences among adults. By contrast, the latent shifting factor showed the highest domain-specific (unique) variance that could not be accounted for by the general factor. Therefore, the correlated factor model exhibited a moderate correlation between shifting and other cognitive abilities (ranging from *r* = .33 to *r* = .49). Nevertheless, this hierarchical model may be conceptually appealing, as the surface characteristics (e.g., attentional control phenomena) are first exposed by the indicators of the first-order factors. Hence, this common cognitive variance might be more predictive for higher-order mental processes, as seen in the prediction of reading ability (Christopher et al. 2016).

Even though the two-layer six-factor model (Figure 3) provided an acceptable data description, it was worse compared to the hierarchical model. This additional model expanded the Im-Bolter et al.’s (2006) model without changing the core structure of the original one. Also, we found that the correlation between inhibition and WMC in the two-layer six-factor model was higher than that of the prior work. Moreover, the non-significant paths from inhibition to updating and from WMC to shifting were different from the original study. However, as described by Im-Bolter et al., mental attention capacity is more than a working memory span task and is a good predictor of other cognitive abilities. This was also demonstrated in our model as relational integration and updating were mostly explained by WMC, which underlines that WMC is more than simply a span task. Critically, a systematic comparison of the best EF model (i.e., five-factor correlated model) with the competing model (i.e., two-layer model and hierarchical model) was conducted to bring together different approaches to EF. This comparison demonstrated that the latent EF factors (updating, shifting, relational integration, WMC, and divided attention) tend to show both unity and diversity through correlating strongly but not perfectly (i.e., the correlation among the factors did not approach 1.0), rather than displaying causality as seen in the two-layer six-factor model. This could move forward our understanding of the EF structure.

Finally, this study contributes to the debate on the factor structure of EFs by reanalyzing the data of a previous study (Himi et al. 2019), especially by means of confirmatory factor analysis. Through this procedure, we tested a restricted factor model so that this could reflect factor–indicator correspondence. Furthermore, the use of a hierarchical model in this study allowed us to quantify common versus skill-specific cognitive variance. Thus, the present study added further insights beyond the previous study.

However, it is necessary to address the impact of the rather small sample size of *N* = 202 used for this study. A small sample size limits the power and therefore the generalizability of the model. Also, the limited number of indicators for updating might enhance the measurement error. Therefore, a recommendation for future studies is to include multiple measures and a large sample to cross-validate the model. Moreover, given the presence of a high amount of multicollinearity among the cognitive variables, we were unable to evaluate and compare several theoretical models we deemed interesting.

Taken together, the EF model relating to updating, inhibition, and shifting (Friedman et al. 2016) was reanalyzed using a broad set of cognitive constructs. However, the findings did not support the factorial validity of the definite model, rather representing the elusiveness of the EF tasks (however, without considering verbal Stroop interference). In conclusion, this study might provide a strong framework in defining and measuring EF in psychological research.

## Figures and Tables

**Table 1:** A short description of the tasks.

Tasks	Authors	Task Description	Dependent Variables
**Shifting**	Friedman et al. (2016)		
Number–letter		When a number–letter pair appears in the top half of the matrix, participants have to classify the number as odd or even; but when the pair appears in the bottom half of the matrix, they should classify the letter as vowel or consonant.	Switch cost: the difference between the mean reaction time (RT) of correct switch trials and the mean RT of correct repeat (nonswitch) trials in random mixed blocks
Color–shape		Participants need to classify the color (green vs. red) or the geometric shape (circle vs. triangle) of the target stimulus.	
Category switch		Participants are instructed to switch back and forth regarding the dimension of animacy (living or nonliving) or size of the target stimulus (smaller or larger than a soccer ball).	
**Updating**	Friedman et al. (2016)		
Keep track		Participants remember the last exemplar of each of the five target categories.	Accuracy (i.e., the proportion of correct trials)
Letter memory		Participants remember the last four letters in the letter string.	
Nonverbal *n*-back	Schellig et al. (2011)	Participants identify the stimulus if the stimulus matches the stimulus *n*-times back.	The average of the z-scores across the 2-back and 3-back tasks
**Inhibition**	Friedman et al. (2016) Kaiser et al. (2010)		
Antisaccade		Participants have to look in the opposite direction of visual cues to detect a briefly presented target.	The proportion of correct target discrimination responses across three antisaccade blocks.
Stop signal		Participants have to categorize and respond to stimuli until a stop signal appears for withholding a response.	The mean stop signal delay is subtracted from the median RT on go trials
**WMC**	Oswald et al. (2015)		
Operation span		Participants have to solve a series of math problems while remembering letters in correct serial order.	The partial-credit score
Reading span		Participants have to identify whether the sentences are meaningful while remembering letters in correct serial order.	
Symmetry span		Participants have to identify whether the patterns are symmetrical while remembering the correct presentation order of red squares in the 4×4 matrix.	
**Relational integration**	von Bastian and Oberauer (2013)		The dependent variable is the discriminability index (d′), reflecting the sensitivity of target detection. It is computed by relating the hit rate and false alarm rate (d′ = z (hit rate)—z (false alarm rate)).
Numerical version		Participants have to respond when three identical last digits appear either in a row, column, or diagonal line in a 3 × 3 matrix.	
Verbal version		Participants are asked to respond when three rhyming words are shown either in a row, column, or diagonal line within the 3 × 3 matrix.	
Figural version		Participants are asked to respond when four black dots form a square in a 3 × 3 matrix.	
**Divided attention**	Sturm (2008)		
Unimodal version		Participants have to monitor two visual stimulus presentation conditions. Whenever the same shape (either square or circle) gets noticeably lighter twice in a row, participants should respond.	The logarithmic mean RT of the given responses
Crossmodal version		Participants are required to monitor one visual and one auditory stimulus presentation conditions. Whenever the square gets noticeably lighter or the sound gets noticeably softer twice in a row, participants are asked to respond.	

**Table 2:** Means, standard deviations (SD), and reliability estimates of the measures.

Tests	Mean	*SD*	Skewness	Kurtosis	Reliability
**Executive functioning**					
**Shifting**					
Number–letter	457.88	157.64	−0.75	0.25	.89 ^c^
Color–shape	828.06	275.50	−0.80	0.92	.92 ^c^
Category switch	592.94	186.20	−0.96	1.30	.83 ^c^
**Updating**					
Keep track	0.75	0.10	−0.68	0.20	.72 ^b^/.73 ^d^
Letter memory	0.69	0.19	−0.41	−0.37	.59 ^b^/.59 ^d^
Nonverbal *n*-back					
Nonverbal 2-back ^a^	1.27	0.10	−0.37	0.73	.84 ^b^
Nonverbal 3-back ^a^	1.22	0.09	−0.36	0.11	.86 ^b^
**Inhibition**					
Antisaccade	0.65	0.17	−0.62	0.03	.94 ^a^
Stop signal	165.93	55.48	0.60	0.25	.94 ^e^
**WMC**					
Operation span	0.82	0.19	−1.39	1.56	.72 ^b^/.73 ^d^
Reading span	0.66	0.23	−0.66	−0.11	.73 ^b^/.73 ^d^
Symmetry span	0.65	0.20	−0.56	−0.03	.55 ^b^/.55 ^d^
**Relational integration**					
Numerical	2.43	0.73	−0.22	−0.13	.77 ^c^
Verbal	2.51	0.71	0.00	−0.31	.72 ^c^
Figural	2.48	0.42	−0.59	0.36	.59 ^c^
**Divided attention**					
Unimodal	481.60	151.06	−1.36	2.07	.96 ^b^
Crossmodal	492.11	171.42	−0.82	0.39	.96 ^b^

Note. The descriptive statistics were given after trimming ±3*SD*s (see text). Reliability estimates were calculated before trimming. All RT measures (in ms) were reversely coded. ^a^ Scores were arcsine transformed, and then converted into z-scores. ^b^ Cronbach’s Alpha. ^c^ Split-half reliability. ^d^ McDonald’s Omega. ^e^ Reliability for difference scores. WMC = working memory capacity.

**Table 3:** Fit statistics of executive functioning (EF) models.

Model	χ^2^	*df*	CFI	RMSEA	SRMR	AIC	BIC
A. Five-factor EF model	130.10	94	.95	.04	.05	214.10	353.05
B. Hierarchical model	136.44	99	.95	.04	.05	210.44	332.85
C. Two-layer six-factor model	168.42	97	.91	.06	.06	246.42	375.44

Note. SRMR = standardized root mean square residual; RMSEA = the root mean square error of approximation; CFI = the comparative fit index; AIC = Akaike information criterion; BIC = Bayesian information criterion.

## Data Availability

The data file (named ‘Final_Multitasking_Data.sav’) is archived in the OSF repository (https://osf.io/tn6hp/ (accessed on 3 March 2016)).

## Appendix A. Factor Loadings for the Exploratory Factor Analysis of EF Variables

**Table A1:** Factor loadings for the exploratory factor analysis of all EF variables (*n* = 202).

Measures	Factors	
	1	2
Number–letter	.56	
Color–shape	.35	
Category switch	.77	
Keep track		.72
Letter memory	−.19	.59
Nonverbal *n*-back		.63
Antisaccade	.22	.49
Stopsignal		.25
	Correlation	
Factor 1	-	
Factor 2	.34	-

Note. The factor loadings less than .20 are not presented.
